# High-Titer Hepatitis C Virus Production in a Scalable Single-Use High Cell Density Bioreactor

**DOI:** 10.3390/vaccines10020249

**Published:** 2022-02-07

**Authors:** Anna Offersgaard, Carlos Rene Duarte Hernandez, Anne Finne Pihl, Nandini Prabhakar Venkatesan, Henrik Krarup, Xiangliang Lin, Udo Reichl, Jens Bukh, Yvonne Genzel, Judith Margarete Gottwein

**Affiliations:** 1 Copenhagen Hepatitis C Program (CO-HEP), Department of Infectious Diseases, Copenhagen University Hospital–Hvidovre, 2650 Hvidovre, Denmark; anna.offersgaard@regionh.dk (A.O.); carlos.rene.duarte.hernandez@regionh.dk (C.R.D.H.); anne.finne.pihl@regionh.dk (A.F.P.); jbukh@sund.ku.dk (J.B.); 2 Copenhagen Hepatitis C Program (CO-HEP), Department of Immunology and Microbiology, Faculty of Health and Medical Sciences, University of Copenhagen, 2200 Copenhagen, Denmark; 3 Esco Aster Pte Ltd., Singapore 486 777, Singapore; nandini.prabhakar@escoaster.com (N.P.V.); xl.lin@escoaster.com (X.L.); 4 Department of Molecular Diagnostics, Aalborg University Hospital, 9000 Aalborg, Denmark; h.krarup@rn.dk; 5 Bioprocess Engineering, Max Planck Institute for Dynamics of Complex Technical Systems, 39106 Magdeburg, Germany; ureichl@mpi-magdeburg.mpg.de (U.R.); genzel@mpi-magdeburg.mpg.de (Y.G.)

**Keywords:** packed-bed bioreactor, CelCradle™, high cell density cell culture, Huh7.5 cells, whole virus vaccine, inactivated virus vaccine, high-titer HCV production, HCV vaccine development

## Abstract

Hepatitis C virus (HCV) infections pose a major public health burden due to high chronicity rates and associated morbidity and mortality. A vaccine protecting against chronic infection is not available but would be important for global control of HCV infections. In this study, cell culture-based HCV production was established in a packed-bed bioreactor (CelCradle™) aiming to further the development of an inactivated whole virus vaccine and to facilitate virological and immunological studies requiring large quantities of virus particles. HCV was produced in human hepatoma-derived Huh7.5 cells maintained in serum-free medium on days of virus harvesting. Highest virus yields were obtained when the culture was maintained with two medium exchanges per day. However, increasing the total number of cells in the culture vessel negatively impacted infectivity titers. Peak infectivity titers of up to 7.2 log_10_ focus forming units (FFU)/mL, accumulated virus yields of up to 5.9 × 10^10^ FFU, and a cell specific virus yield of up to 41 FFU/cell were obtained from one CelCradle™. CelCradle™-derived and T flask-derived virus had similar characteristics regarding neutralization sensitivity and buoyant density. This packed-bed tide-motion system is available with larger vessels and may thus be a promising platform for large-scale HCV production.

## 1. Introduction

Hepatitis C virus (HCV) is a small enveloped RNA virus of the *Flaviviridae* family with a genome of ~9.6 kilobases encoding a polyprotein processed into 3 structural proteins (Core, envelope glycoproteins E1 and E2) and 7 nonstructural proteins (p7, NS2, NS3, NS4A, NS4B, NS5A and NS5B) [1]. There are 8 major genotypes of HCV differing in around 30% of their nucleotide and amino acid sequence. These are further classified into at least 90 subtypes [2,3,4,5].

HCV infections pose a global health burden and remain endemic in most regions of the world despite the recent development of highly effective direct-acting antiviral (DAA) drugs [6,7]. Around 25% of HCV infections are spontaneously cleared whereas the remaining cases develop into chronic liver inflammation which, over time, increases the risk of serious liver diseases such as cirrhosis and cancer [8]. Worldwide, at least 58 million people are estimated to live with chronic HCV infection. Annually, there are at least 1.5 million new infections and 300,000 HCV-related deaths [6,9,10]. A Global Health Sector Strategy on viral hepatitis announced by the World Health Organization aims to eliminate viral hepatitis as a major public health threat [11], however, with no vaccine available against HCV, it may prove difficult to reach this goal. Although recently developed DAA drugs allow high cure rates of treated individuals, HCV elimination by treatment is hampered by unawareness of infection status among infected individuals, lack of screening programs, and the high costs of the DAAs [7]. Further, there is a risk of re-infection for individuals in high-risk populations, and emerging viral resistance may give rise to reduced treatment efficacy in the future [12]. A vaccine to prevent chronic HCV infection would be an essential tool to decrease the morbidity and mortality caused by this virus [6,7].

While many vaccine strategies have been explored for HCV, there is still no licensed vaccine protecting against chronic HCV infection [7]. The most advanced candidate was a viral vector vaccine inducing T cell responses; however, in a recent phase I/II trial this candidate did not protect against chronic infection [13]. As an alternative approach, B cell vaccine candidates are being studied with great interest. The most advanced candidate is a recombinant E1/E2 vaccine, which induced neutralizing antibodies in a phase I trial, but only in a relatively low percentage of vaccinees [14,15]. Many successful viral vaccines are based on whole virus particles inducing neutralizing antibodies [16,17,18]. Notably, when immunizing mice, whole inactivated HCV particles seemed more immunogenic than recombinant envelope proteins [19]. Further, protective neutralizing antibodies in infected individuals have been shown to target conformational epitopes of the HCV envelope proteins [14,20,21], highlighting the importance for a B cell vaccine to present E1E2 heterodimers that closely resemble the native conformation. An inactivated whole virus vaccine could thus be an attractive approach in HCV vaccine development.

In the first few decades following the discovery of HCV, no cell culture system supporting the full virus life cycle with production of infectious particles was available [3]. In 2005, the first cell culture system for HCV was developed based on a single patient isolate of genotype 2a, JFH1, and human hepatoma (Huh) 7-derived cell lines [22,23]. Subsequently, culture systems producing HCV of other genotypes and isolates were developed, mostly depending on cell culture adaptive viral mutations [3]. However, originally developed recombinants yielded relatively low infectivity titers, between 3–5 log_10_ focus forming units (FFU)/mL, which is considered suboptimal for vaccine development [22,24,25,26,27]. Using a serial passage approach in Huh7.5 cells, a further adapted 5a(SA13)/2a(JFH1) recombinant was developed yielding peak infectivity titers of approximately 6 log_10_ FFU/mL [27,28]. 

Vaccines based on whole virus particles as well as research applications such as ultrastructural studies and immunogenicity studies require high-titer virus production. Packed-bed bioreactors support adherent cell cultures and are attractive alternatives to monolayer cell culture-based production of HCV particles [29]. Compared to monolayer cultures, packed-bed bioreactors allow increased cell densities and improved control of cell culture process parameters, which may enhance virus yields [30,31]. We previously obtained proof-of-concept that HCV could grow to high infectivity titers of up to 7.6 log_10_ FFU/mL in a hollow fiber bioreactor; however, this hollow fiber bioreactor system was not scalable [32].

The CelCradle™ bioreactor (Esco Aster Pte. Ltd., Singapore, Singapore) was previously reported to support efficient production of Japanese encephalitis virus [33], hepatitis D virus-like particles [34], different strains of influenza A virus [35], severe acute respiratory syndrome coronavirus-2 (SARS-CoV-2) [36], as well as a number of viruses relevant for veterinary vaccine purposes [37]. It is a packed-bed bioreactor using BioNOCII™ macrocarriers and it employs a tide-motion principle, thereby conferring minimal shear stress to cells during medium circulation. 

In this study, high-titer serum-free (SF) HCV particle production was established in the single-use packed-bed CelCradle™ bioreactor. Being a laboratory-scale version of the larger linearly scalable high-density TideXCell systems, which are compatible with good manufacturing practices (GMP) [38], this system may be attractive for applications requiring large quantities of HCV viral particles.

## 2. Materials and Methods

### 2.1. Maintenance of Huh7.5 Cells

Huh7.5 cell cultures were maintained in cell culture T flasks in serum-containing medium (SCM): Dulbecco’s Modified Eagle Medium (DMEM) (Gibco, Paisley, UK) containing 4 mM GlutaMAX and supplemented with 10% fetal bovine serum (Sigma, St. Louis, MO, USA), 100 U/mL penicillin and 100 µg/mL streptomycin (Sigma). In T175 flasks (Nunc, Roskilde, Denmark) and triple layer culture flasks (Nunc), Huh7.5 cells were passaged every 2–3 days. All cell cultures were maintained at 37 °C and 5% CO_2_.

### 2.2. HCV Virus Stocks

Two sequence-confirmed stocks of the genotype 5a recombinant SA13/JFH1_core-NS5B_ [28], grown in monolayer Huh7.5 cell cultures in T flasks, were used in this study. A third passage virus stock with an infectivity titer of 5.8 log_10_ FFU/mL, grown under serum-containing (SC) conditions [39], was used to inoculate CelCradle™ cultures, shake flask cultures, and for virus characterization experiments. A fourth passage virus stock with an infectivity titer of 5.1 log_10_ FFU/mL [32], grown under SF conditions [39], was used for virus characterization experiments.

### 2.3. CelCradle™ Culture and Virus Production

#### 2.3.1. Cell Seed, Attachment, and Expansion in the CelCradle™

For CelCradle™ cultures, CelCradle™ 500AP bottles (Esco Aster Pte. Ltd.) with a working volume of 500 mL were used. The standard carrier bed of 500AP bottles is 100 mL and comprises 5.5 g BioNOCII™ carriers (~15.5 cm^2^ per carrier and ~850 carriers per bottle; total surface ~13,200 cm^2^ per bottle [38,40]). In the experiment with a carrier bed of roughly 200 mL and 10.7 g BioNOCII™ carriers, additional carriers were autoclaved (121 °C, 30 min) in phosphate buffered saline (PBS) (Sigma) and added to the 500AP bottle.

The bioreactor is operated by a tide-motion principle for cycles of alternating cell culture nutrition and gas exchange.

Huh7.5 cells were expanded in triple layer culture flasks under SC conditions. Cells were harvested and centrifuged at 200 g for 5 min and prepared in a total volume of 120 mL SCM. All experiments were initiated with a cell seeding density of 2 × 10^5^ cells/carrier, with exception of the culture with 10.7 g BioNOCII™ carriers seeded at a slightly lower seeding density of 1.7 × 10^5^ cells/carrier.

During attachment, the 500AP bottle was incubated upside down at 37 °C and 5% CO_2_ using a cap without membrane. The bottle was swirled every 30–60 min. One to 3 h after seeding, cells remaining in suspension were counted to evaluate attachment efficiency as:

attachment efficiency = (1 − (total cells in suspension/total cells seeded))*100(1)

When the attachment efficiency was >85%, medium was added to a total of 500 mL, and the bottle was mounted in the CelCradle™ stage with the following settings: rising rate 1.5 mm/s, top holding time 30 s, downwards rate 1.5 mm/s, bottom holding time 30 s.

#### 2.3.2. HCV Infection of the CelCradle™ Culture in SCM

HCV infection of CelCradle™ cultures was carried out when the carrier specific glucose consumption rate (csGCR, for definition see below) was 0.002–0.004 mmol/carrier/day, as specified for individual experiments. Cell culture medium was replaced with 300 mL fresh medium prior to infection. A virus inoculum (Section 2.2) corresponding to an MOI of 0.006 according to the number of seeded cells was used for infection in all experiments. Thus, cultures with a carrier bed of 5.5 g BioNOCII™ carriers were inoculated with 1.1 × 10^6^ FFU diluted in 50 mL cell culture medium. The culture with a carrier bed of 10.7 g BioNOCII™ carriers was inoculated with 2 × 10^6^ FFU diluted in 50 mL cell culture medium. Following inoculation, the CelCradle™ stage settings were changed for 3 h: rising rate 1.5 mm/s, top holding time 4 min 30 s, downwards rate 1.5 mm/s and bottom holding time 0 s. Subsequently, cell culture medium was added to a total of 500 mL and CelCradle™ stage settings were returned to culture settings, as described above.

#### 2.3.3. Virus Harvests under SF Culture Conditions

To obtain SF virus harvests, cultures were maintained in SF Adenovirus Expression Medium (AEM, Gibco) with 100 U/mL penicillin and 100 µg/mL streptomycin (Sigma) from the time where ≥80% of cells were estimated to be infected according to immunostaining. For the culture with a carrier bed of 10.7 g BioNOC™ the serum-free medium (SFM) was additionally supplemented with 4 mM GlutaMAX (Thermo Fisher Scientific, Waltham, MA, USA). To do the exchange, SCM was removed, cells were washed sequentially with 1 × 500 mL PBS and 1 × 500 mL SFM and cultured in fresh 500 mL SFM. After changing to SF conditions, virus containing supernatant was harvested once or twice per day removing ~90% of the supernatant volume and replacing it with fresh medium (as specified for individual experiments depending on the daily medium exchange (DME) frequency). Harvests were stored at −80 °C. CelCradle™ cultures were terminated following confirmation that HCV infectivity titers had peaked and declined to ~6 log_10_ FFU/mL.

#### 2.3.4. Maintenance and Monitoring of the CelCradle™ Cultures and Evaluation of Spread of Infection

Cell culture medium was exchanged and/or supplemented with 45% glucose solution (Sigma) and 8% NaHCO_3_ solution (Sigma) 1–3 times per day, as specified for individual experiments, aiming to maintain glucose levels above 11 mM and pH above 7.0. Sampling of culture medium and measurement of glucose, pH (and in selected experiments lactate, glutamine and ammonia) were done both before and after each DME or adjustment of glucose or pH levels, resulting in up to six measurements per day. The pH value was measured with a FiveGo F2 pH meter (Mettler Toledo, Columbus, OH, USA). Glucose concentrations were measured with a handheld glucometer (Accu-Check Compact Glucometer, Roche, Mannheim, Germany) or with a bioanalyzer (Cedex Bio Analyzer, Roche). Additional parameters, lactate, glutamine (the measurements represent free glutamine and glutamine available as GlutaMAX), and ammonia, were evaluated in selected experiments with the Cedex Bio Analyzer. Glucose consumption between two time points was calculated as the difference in the total amount of glucose available in the culture medium at two consecutive time points of sampling. All glucose consumption values obtained for each day were summed to obtain the respective glucose consumption rate (GCR). The calculated GCR was divided by the number of carriers in the culture for csGCR. Glutamine consumption was calculated as described for glucose consumption.

Cells per carrier were counted every day on early culture days and then less frequently as specified for individual experiments. At each timepoint, five carriers were collected, washed with PBS and incubated with trypsin-EDTA solution (Sigma) or Accumax solution (Sigma) at 37 °C for cell detachment. Detached cells were collected in DMEM with 20% fetal bovine serum and counted with a Scepter™ 2.0 Cell Counter (Merck, Darmstadt, Germany) or in a hemocytometer with Trypan Blue (Sigma). To evaluate detachment efficiency, carriers were fixed with 70% ethanol for 5 min followed by 99% ethanol for 5 min; cell nuclei were stained with Hoechst 33342 (Molecular Probes, Eugene, OR, USA). As a control, a non-trypsinized carrier was similarly fixed and stained. 

The percentage of HCV infected cells in the CelCradle™ cultures was evaluated by seeding cells detached for cell counting in a chamber slide followed by immunostaining for HCV NS5A, as described below. Virus production was evaluated by determining HCV infectivity titers, as described below.

In this study, high-yield culture days were defined as days with a virus yield of at least 9.2 log_10_ FFU, corresponding to the total number of FFU in 450 mL with an infectivity titer of ≥6.5 log_10_ FFU/mL. Moderate-yield culture days were defined as days with a yield of at least 8.7 log_10_ FFU and <9.2 log_10_ FFU; ≥8.7 log_10_ FFU correspond to the total number of FFU in 450 mL with an infectivity titer of ≥6 log_10_ FFU/mL. Total accumulated FFU were calculated summing up the number of FFU obtained in each harvest, based on determined HCV infectivity titers and harvest volumes of 450 mL. Cell specific virus yield (CSVY) was calculated from the accumulated FFU and the peak total cell numbers measured during the time of culture as FFU/cell.

### 2.4. Shake Flask Cell Cultures

BioNOCII™ carriers were washed with PBS and autoclaved (121 °C, 30 min). Sixty carriers (~0.39 g) were prepared in each 250 mL shake flask (Corning, Corning, NY, USA) and pre-incubated with cell culture medium, which was removed upon addition of cells. Cell seed, cell expansion, and infection were carried out in SCM. A cell seed of 2 × 10^5^ cells/carrier was prepared in 8 mL cell culture medium per flask and added to the carriers. The flasks were swirled every 30–60 min for 3 h when attachment was evaluated, as described above. Cell culture medium was subsequently added for a total volume of 0.6 mL/carrier and shake flasks were placed on a rocking shaker (VariMix Platform Rocker, Thermo Scientific, 10 rpm). 

Shake flask cultures were infected when the GCR was 0.002 mmol/carrier/day with an inoculum (Section 2.2) of 6.8 × 10^4^ FFU in 10 mL cell culture medium. Cultures were incubated for 3 h without shaking. Subsequently, cell culture medium up to 0.6 mL/carrier was added and shaking was resumed. For virus production under SF conditions, cultures were maintained in SFM from the time where ≥80% of cells were estimated to be infected according to immunostaining. SCM was removed, the culture was washed once with PBS and once with SFM and cells were subsequently cultured in SFM. 

The shake flask cultures were maintained as described for the CelCradle™ cultures with DME and/or supplementation once or twice per day, as specified with sampling before and after each DME and/or glucose and pH adjustments. Different DME frequencies were initiated when daily glucose consumption exceeded the amount of glucose supplied by one DME. Three carriers were collected for cell counting as described for CelCradle™ cultures. 

The percentage of HCV infected cells, HCV infectivity titers, and CSVY were evaluated as described for CelCradle™ cultures.

### 2.5. Immunostaining of HCV NS5A

The percentage of HCV infected cells in Huh7.5 cell cultures was evaluated by immunostaining for HCV antigen [39,41]. Infected cells were seeded in a chamber slide (Thermo Fisher Scientific) and the next day fixed with ice-cold acetone (Merck). Cells were stained with primary antibody 9E10 targeting HCV NS5A [22] diluted 1:3000 in PBS with 1% bovine serum albumin (Roche) (*w/v*) and 0.2% skimmed milk (Easis, Aarhus, Denmark) (*w/v*) and secondary antibody Alexa Fluor 488 goat anti-mouse IgG (Invitrogen, Waltham, MA, USA) diluted 1:500 in combination with Hoechst 33342 diluted 1:1000 in PBS-Tween (PBS with 0.1% (*v/v*) Tween-20 (Sigma)).

### 2.6. HCV Infectivity Titers, RNA Titers, and Core Titers

HCV infectivity titers in the cell culture supernatant were determined, as described previously [42]. In brief, Huh7.5 cells were seeded in a 96-well plate with 6.5 × 10^3^ cells/well the day prior to infection. Cells were infected with virus dilutions in triplicates, fixed 48 h after infection with ice-cold methanol (J. T. Baker, Radnor, PA, USA), treated with 3% H_2_O_2_ and immunostained with primary antibody 9E10 targeting HCV NS5A [22] diluted 1:5000 in PBS with 1% bovine serum albumin (*w/v*) and 0.2% skimmed milk (*w/v*) and secondary antibody ECL Anti-mouse IgG Horseradish Peroxidase linked from sheep (Amersham Biosciences, Marlborough, MA, USA) diluted 1:500 in PBS-Tween. FFU were visualized with Bright-DAB solution kit (Immunologic, Duiven, The Netherlands), imaged and automatically counted for quantification, as described using an Immunospot series 5 UV analyzer (CTL Europe GmbH, Bonn, Germany) [42,43]. Infectivity titers were calculated as FFU/mL, as described [42]. HCV infectivity titers are means of triplicates and error bars presented in figures represent the standard errors of the means (SEM).

HCV RNA titers (international units (IU)/mL) in cell culture supernatant were determined using a quantitative reverse-transcription polymerase chain reaction, as described previously [41]. 

HCV Core titers in cell culture supernatants were determined with the ARCHITECT HCV Ag assay (Abbott, Chicago, IL, USA), as previously described [44].

The specific infectivity was calculated for selected harvests as FFU/mL divided by IU/mL or as FFU/mL divided by amol Core/mL.

### 2.7. HCV Neutralization Assays

Cell culture-based in vitro HCV neutralization assays were carried out as described previously [27]. Briefly, 6.5 × 10^3^ cells/well were plated in 96-well poly-D-lysine coated plates (Nunc) the day prior to infection. 20–110 FFU/well were incubated with antibody, C211 [45], AR3A [46] or AR4A [47], serially diluted in SCM and incubated for 1 h at 37 °C; each antibody dilution was tested in triplicate. Virus-antibody mixes were transferred to cells and incubated for 3 h at 37 °C. Cells were subsequently washed with PBS and incubated with SCM. After 48 h, cells were fixed, stained, and FFU counts were obtained, as described for HCV infectivity titrations above. At least six virus-only wells were included as positive controls, and at least six wells without virus were included as negative controls. The percentage of neutralization for each well was calculated by relating the FFU count in each well to the mean FFU count in the virus-only wells. EC_50_ values were calculated in GraphPad prism version 8 fitting variable-slope sigmoidal dose-response curves:

y = bottom + (top − bottom)/(1 + 10^((logEC_50_-x) * hillslope)) (2)

with bottom and top constraints of 0 and 100, respectively. The presented error bars show SEM.

### 2.8. Equilibrium Density Gradient Centrifugation

The buoyant density of virus particles was determined as described previously [48]. Briefly, OptiPrep Density Gradient Medium (Sigma) was diluted in PBS for 40%, 30%, 20% and 10% solutions, which were added on top of each other in Beckman centrifuge tubes, 2.5 mL each, and incubated overnight at 4 °C for formation of continuous gradients. 1 mL virus containing cell culture supernatant was layered on the gradient and subjected to ultracentrifugation in a Beckman XL-70 centrifuge with an SW41 rotor at 35,000 rpm for 18 h at 4 °C. Eighteen fractions of 550 µL were collected from the bottom of the tube. The density of each fraction was determined, and the infectivity titer of each fraction was obtained. The percentage of the total FFU present in each fraction was calculated to determine the relative recovery per fraction.

### 2.9. Virus Genome Sequencing

RNA was extracted and the open reading frame was amplified by reverse transcription and polymerase chain reaction with sequence-specific primers, as described previously [49,50]. The full open reading frame was sequenced by Sanger sequencing (Macrogen Europe B.V., Amsterdam, the Netherlands).

## 3. Results

### 3.1. Attachment and Expansion of Huh7.5 Cells Cultured on BioNOCII™ Carriers

Small-scale pilot experiments carried out with BioNOCII™ carriers in culture dishes demonstrated efficient attachment and expansion of Huh7.5 cells on these carriers. Furthermore, Huh7.5 cells cultured on BioNOCII™ carriers were susceptible to HCV infection and could be maintained under SF conditions after cell expansion (data not shown). An initial CelCradle™ culture was seeded with 2 × 10^5^ cells/carrier in SCM with inversion of the CelCradle™ bottle during attachment. Attachment efficiencies found for this initial experiment and across all experiments reported here were 87–96% after 1–2 h of incubation (Figure S1). In this first experiment, medium was exchanged once every day from the third day post cell seeding (dpcs). Cell numbers exceeding 1 × 10^6^ cells/carrier were obtained 5 dpcs and the total number of cells in the bottle peaked at 9 dpcs with 1.6 × 10^9^ cells; glucose consumption rates were up to 9 mmol/day (Table 1).

### 3.2. Production of HCV in SCM (CC-DMEx1-SCM)

Attachment and growth of Huh7.5 cells are best supported in SCM, while SF conditions appear not to support efficient HCV infection and spread of infection [39]. Therefore, HCV production was first tested in SCM. Furthermore, according to observations in T flasks, spread of HCV infection is most efficient in sub-confluent cell layers. The culture was therefore infected at 3 dpcs, when the csGCR was 0.004 mmol/carrier/day, to allow simultaneous propagation of cells and virus infection. In addition, for this approach a smaller virus inoculum was required compared to infection at the time of peak cell numbers. An inoculum of 1.1 × 10^6^ FFU was used in all experiments with a standard carrier concentration. 

**Figure 1:**
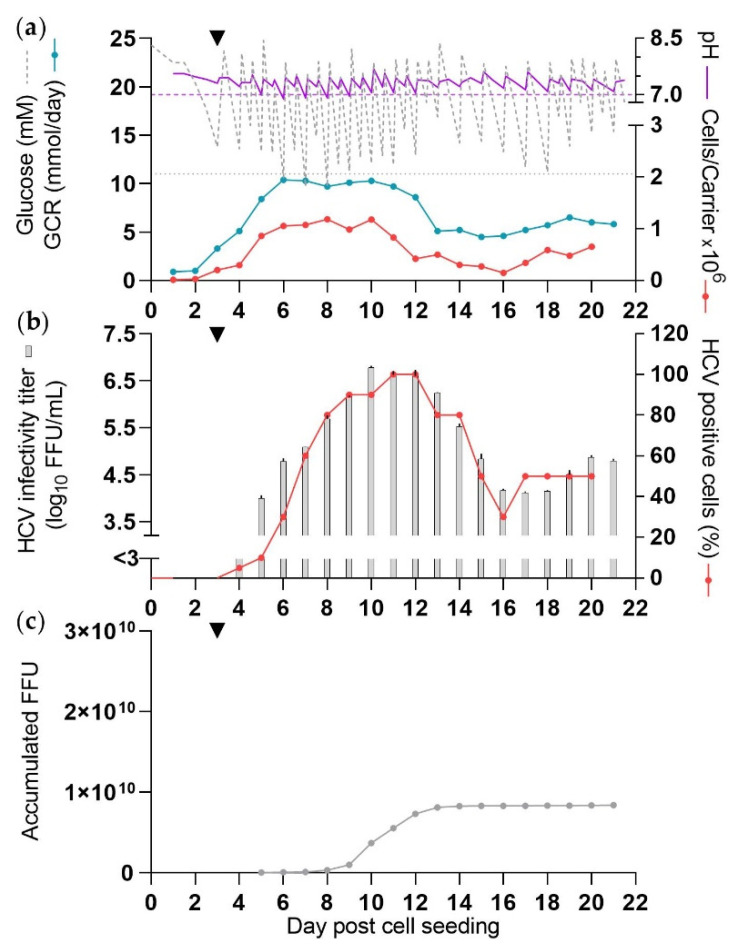
Production of HCV in SCM (CC-DMEx1-SCM). The culture was seeded with 2 × 10⁵ cells/carrier in SCM and infected with 1.1 × 10⁶ FFU at 3 dpcs (black arrow). Purple dashed line: pH 7, grey dotted line: 11 mM glucose. From 3 dpcs medium was exchanged each morning. At 4–12 dpcs glucose and/or NaHCO_3_ solution was added as required upon DME and in the evening. Glucose concentration and pH were measured up to four times per day, each time before and after DME and/or addition of glucose and/or NaHCO_3_ solution. Carriers were sampled daily for cell count. (**a**) Glucose concentration, pH, glucose consumption rate (GCR), and cells/carrier. (**b**) HCV infectivity titers shown as means of triplicates with standard errors of the means (SEM) and percentage HCV NS5A positive cells. (**c**) Accumulated focus forming units (FFU) calculated according to harvest volumes of 450 mL.

Throughout the experiment, one DME was carried out, mostly in the morning. In the evening, glucose was supplemented and pH adjusted, aiming to maintain glucose concentrations >11 mM and pH >7.0. During the experiment, glucose concentrations were in the range of 10–25 mM and pH was in the range of 6.9–7.7 (Table 1, Figure 1a). 

High cell numbers (exceeding 1 × 10^6^ cells/carrier) were observed from 6–10 dpcs with a peak total cell number of 0.96 × 10^9^ at 8 dpcs and GCR increased to ~10 mmol/day at 6–11 dpcs (Figure 1a). From 12 dpcs cell numbers declined below 0.6 × 10^6^ cells/carrier.

At least 80% of cells were estimated to be infected with HCV at 8 dpcs and the percentage of infected cells appeared to drop below 80% from 15 dpcs (Figure 1b). There were three high-yield culture days (10–12 dpcs) considered to be days with production of ≥9.2 log_10_ FFU/day, and a total of five culture days (9–13 dpcs) with at least a moderate-yield, considered to be days with production of ≥8.7 log_10_ FFU per day. The peak HCV infectivity titer was 6.8 log_10_ FFU/mL at 10 dpcs (Figure 1b). A total of 0.81 × 10^10^ FFU were harvested from this culture until 13 dpcs, which was the last moderate-yield culture day. Remaining days contributed <2% per day to the total yield (Figure 1c). CSVY of the culture based on the amount of accumulated FFU by 13 dpcs and the peak total cell number was 8.5 FFU/cell (Table 1).

Considering the course of the experiment, the cell numbers peaked prior to the peak in infectivity titers and quickly declined once a large proportion of cells were estimated to be infected. The high-yield culture days were two days delayed compared to the time when ≥80% infected culture cells were first observed, and infectivity titers and the percentage of infected cells declined simultaneously. 

### 3.3. Production of HCV in SFM (CC-DMEx1-SFM_1_)

For production of HCV in SFM, cells were initially seeded and infected in SCM, as described above. From the time when ≥80% of the culture was estimated to be infected SFM was used. To further optimize the time of infection in comparison to CC-DMEx1-SCM, aiming to achieve high cell numbers and ≥80% infection at the same time, this culture (CC-DMEx1-SFM_1_) was inoculated with HCV when the csGCR was 0.002 mmol/carrier/day at 2 dpcs. 

**Figure 2:**
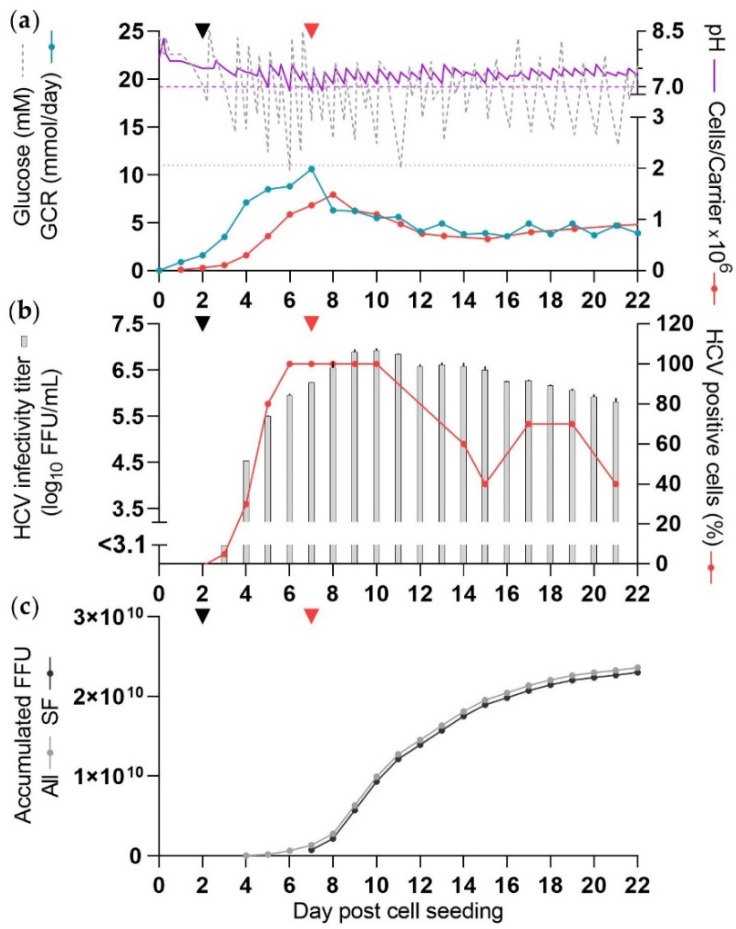
Production of HCV in SFM (CC-DMEx1-SFM_1_). The culture was seeded and infected at 2 dpcs (black arrow), as described in Figure 1. Purple dashed line: pH 7, grey dotted line: 11 mM glucose. At 3 dpcs medium was exchanged in the evening and from 4 dpcs medium was exchanged each morning. From 4 dpcs glucose and/or NaHCO_3_ solution was added as required and glucose concentration and pH were measured as described in Figure 1. Carriers were sampled daily until 13 dpcs and then less frequently. From 6 dpcs (evening) the culture was maintained in SFM (red arrow). (**a**) Glucose concentration, pH, GCR, and cells/carrier. (**b**) HCV infectivity titer and percentage HCV NS5A positive cells. (**c**) Accumulated FFU, calculated according to harvest volumes of 450 mL.

The culture was maintained as CC-DMEx1-SCM regarding DME, glucose concentration- and pH adjustments, and similar ranges of glucose concentrations and pH values were measured (Table 1, Figure 2a). 

Prior to DME at 6 and 11 dpcs, glucose levels were just below the desired lower limit, and pH was below the target range at 6 and 7 dpcs.

The cell expansion was as observed in the previous experiment with a slightly higher peak total cell number of 1.2 × 10^9^ cells at 8 dpcs (Figure 2a) followed by a decline. GCR peaked at 11 mmol/day at 7 dpcs and dropped following the change to SFM. 

The percentage of infected cells was estimated to be ≥80% from 6 dpcs to at least 10 dpcs (Figure 2b). From the evening at 6 dpcs the culture was maintained under SF conditions and virus was harvested once per day upon DME starting 7 dpcs. During this SF virus production period there were eight high-yield culture days at 8–15 dpcs, and a total of 13 days with at least a moderate yield, 7–19 dpcs. The peak HCV infectivity titer was 6.9 log_10_ FFU/mL at 9 dpcs (Figure 2b). The total yield of SF virus until 19 dpcs was 2.2 × 10^10^ FFU (Figure 2c). The last moderate yield culture day was 19 dpcs, and remaining days contributed <2% per day to the total HCV yield. CSVY was 18.5 FFU/cell (Table 1). 

With the adjusted time of infection, culture days with high cell numbers and high percentage of infected cells were better synchronized. Overall, the changes in infectivity titers of the virus harvests followed the percentage of infected cells. The reduced cell numbers observed from 10 dpcs did not cause an immediate drop in harvest titers. As observed in the previous experiment, high-yield culture days were two days delayed compared to the time when ≥80% infection was first observed. The virus yield from this culture was ~2.7-fold higher than that from the first culture maintained with SCM throughout the experiment (CC-DMEx1-SCM). 

### 3.4. Increased Cell Numbers Did Not Enhance HCV Production under SF Conditions (CC*-DMEx2-SFM)

In the initial HCV productions, CC-DMEx1-SCM and CC-DMEx1-SFM_1_, total cell numbers in the cultures reached 0.96–1.2 × 10^9^ cells (Table 1). To investigate if increased total cell numbers would improve virus yield additional BioNOCII™ carriers were added to the CelCradle™ bottle for a total of 10.7 g BioNOCII™ carriers (standard: 5.5 g BioNOCII™ carriers). 

**Figure 3:**
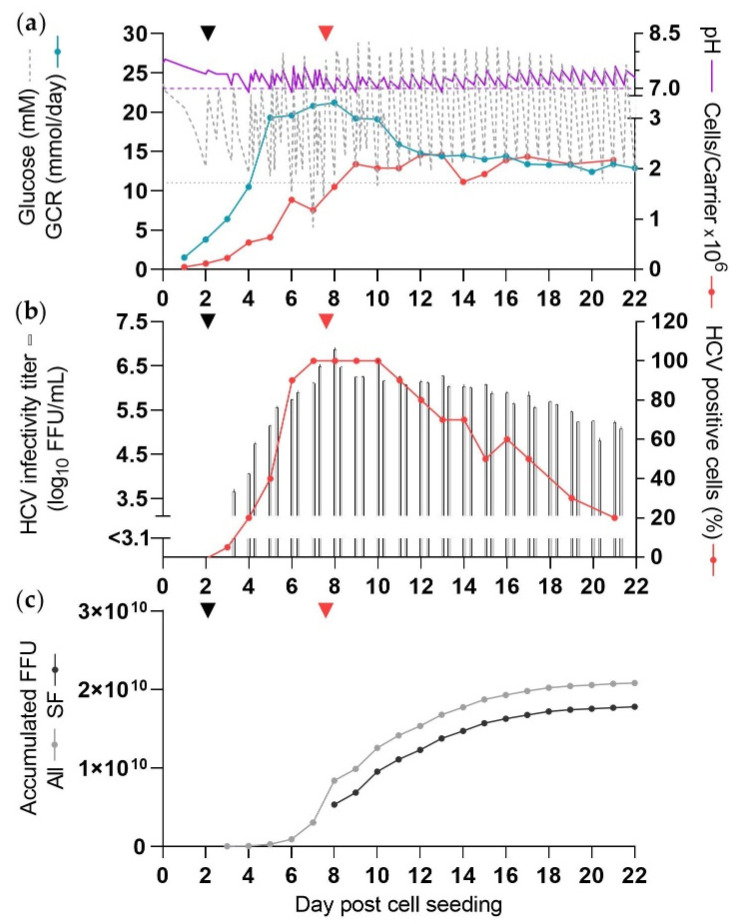
SF HCV production in the CelCradle™ with additional BioNOCII™ carriers for increased cell numbers (CC*-DMEx2-SFM). The culture was seeded with 1.7 × 10⁵ cells/carrier (10.7 g BioNOCII™ carriers) and infected with 2 × 10⁶ FFU at 2 dpcs (black arrow), as described in Figure 1. Purple dashed line: pH 7, grey dotted line: 11 mM glucose. At 2 and 3 dpcs medium was exchanged in the evening and from 4 dpcs medium was exchanged each morning and evening. Glucose and/or NaHCO_3_ solution was added upon DME as required, and at 5–16 dpcs in the morning, afternoon and evening. Glucose concentration and pH were measured up to six times per day, each time before and after DME and/or addition of glucose and NaHCO_3_ solution. Carriers were sampled daily until 17 dpcs and then less frequently. From 7 dpcs the culture was maintained in SFM (red arrow). (**a**) Glucose concentration, pH, GCR, and cells/carrier. (**b**) HCV infectivity titers (two per day) and percentage HCV NS5A positive cells. (**c**) Accumulated FFU calculated according to harvest volumes of 450 mL.

As for CC-DMEx1-SFM_1_, infection was carried out at a csGCR of 0.002 mmol/carrier/day at 2 dpcs (Figure 3a). In general, throughout the experiment two times DME with additional adjustment of glucose concentration and pH values were required. Glucose concentrations were in the range of 5–29 mM and pH was in the range of 6.9–7.9 (Figure 3a, Table 1). Lactate, glutamine and ammonia concentrations were measured in this and subsequent experiments. Lactate concentrations were up to 48 mM prior to DME at 7 dpcs, and subsequently decreased to lower levels while the culture was maintained in SFM (Figure S2). In this experiment, glutamine was supplied in SFM (as GlutaMAX). Glutamine consumption peaked at 1.6 mmol/day at 7 dpcs and subsequently declined to insignificant levels from 12 dpcs. Ammonia concentrations peaked at 2 and 7 dpcs at around 1 mM, and as reflected by glutamine consumption, ammonia concentrations were very low or undetectable from 12 dpcs (Figure S2).

A total cell number of 3.6 × 10^9^ cells was reached at dpcs 12 (Figure 3a), which was 3-fold higher compared to CC-DMEx1-SFM_1_. The GCR was approximately doubled compared to the cultures with standard carrier concentration and peaked at 23 mM at 7 dpcs. 

As for CC-DMEx1-SFM_1_, the percentage of infected cells was estimated to be ≥80% at 6–12 dpcs (Figure 3b). The culture was maintained under SF conditions with two virus harvests per day from 7 dpcs. There were 6 high-yield days (8–13 dpcs) and 11 culture days with at least a moderate yield (8–18 dpcs) under SF conditions (Figure 3b,c). The peak infectivity titer was 6.9 log_10_ FFU/mL at 8 dpcs, similar to that of CC-DMEx1-SFM_1_. The total yield by 18 dpcs was 1.7 × 10^10^ FFU (Figure 3c). The last moderate-yield culture day was 18 dpcs, and remaining days contributed only <2% per day to the total yield. CSVY was 4.8 FFU/cell (Table 1).

Overall, the total yield from CC*-DMEx2-SFM with increased total cell numbers was about 20% lower than that of the previous SF cultivation, CC-DMEx1-SFM_1_, and CSVY was reduced by ~4 fold (Table 1).

### 3.5. Evaluation of the Influence of DME Frequency on HCV Yield in Shake Flasks (ShF-DMEx1-SCM, ShF-DMEx2-SCM, ShF-DMEx1-SFM, ShF-DMEx2-SFM)

In the previous experiment, increasing the total cell numbers in the culture led to a reduced HCV yield and a low CSVY. As a different approach to enhance virus yield, we evaluated different DME frequencies. To this end, we established a scale-down model in 250 mL shake flasks with the same ratio of medium volume per carrier as in a CelCradle™ culture with standard carrier concentrations. Yields were compared for cultures maintained in either SCM or SFM with either one (ShF-DMEx1-SCM and ShF-DMEx1-SFM) or two DME (ShF-DMEx2-SCM and ShF-DMEx2-SFM). In addition, glucose and pH levels were adjusted in all cultures up to twice per day. Infection was carried out at a csGCR of 0.002 mmol/carrier/day at 1 dpcs with an inoculum of 6.8 × 10^4^ FFU HCV.

**Figure 4:**
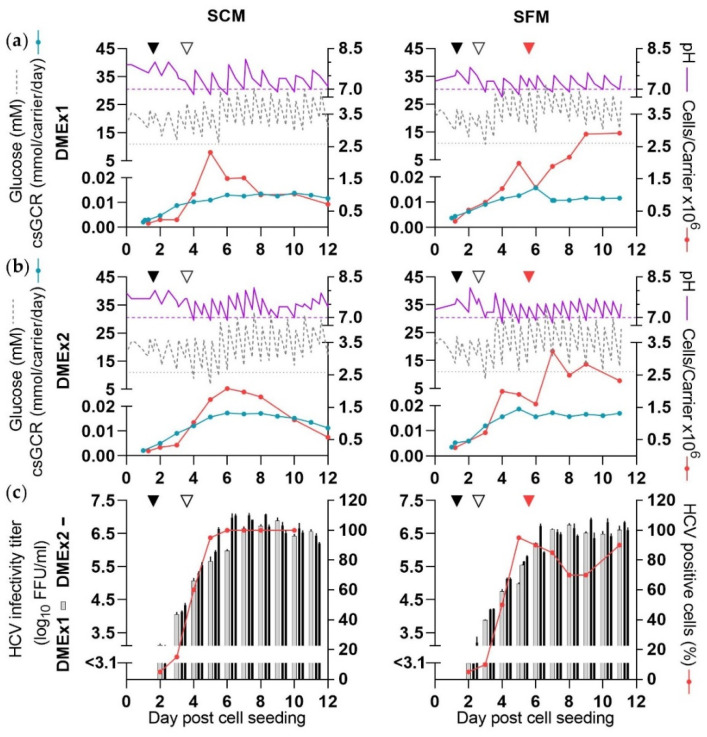
Evaluation of the influence of medium exchange frequency on HCV yields in shake flasks (ShF-DMEx1-SCM, ShF-DMEx2-SCM, ShF-DMEx1-SFM, ShF-DMEx2-SFM). The cultures were seeded with 2 × 10^5^ cells/carrier and infected with 6.8 × 10^4^ FFU at 1 dpcs (black arrow). Purple dashed line: pH 7, grey dotted line: 11 mM glucose. Different DME frequencies, (**a**) DMEx1, morning versus (**b**) DMEx2, morning and evening, were initiated at 3 dpcs or 2 dpcs in left and right panels, respectively (open arrow). Glucose and/or NaHCO_3_ solution was added, glucose concentration and pH were measured, and carriers were sampled as described in Figure 1. In the indicated cultures SF conditions were applied from 5 dpcs (red arrow). (**a**,**b**) Glucose concentration, pH, csGCR, and cells/carrier. (**c**) HCV infectivity titers and percentage HCV NS5A positive cells; the curve represents both cultures as identical values were recorded throughout the experiment.

Under SC conditions the two different DME frequencies were applied from 3 dpcs. Aiming at glucose concentrations of >11 mM, shake flask glucose concentrations had to be adjusted to higher levels than in the CelCradle™ cultures. pH values were maintained in a range similar to that in the CelCradle™ cultures; however, as experienced in the CelCradle™ experiments, pH values dropped below 7 on several culture days (Figure 4a,b, Table 2). 

In both ShF-DMEx1-SCM and ShF-DMEx2-SCM, similar peak cell numbers of ~2 × 10^6^ cells/carrier were reached (Figure 4a,b). 

In both cultures, ≥80% infection was reached 4 days post infection, as also seen for the CelCradle™ experiments infected at a csGCR of 0.002 mmol/carrier/day. In ShF-DMEx1-SCM high-yield culture days were at 7–11 dpcs with a peak titer of 6.9 log_10_ FFU/mL. ShF-DMEx2-SCM had two more high-yield culture days and a peak titer of 7.1 log_10_ FFU/mL (Figure 4c, Table 2). CSVY was ~4-fold increased in ShF-DMEx2-SCM as compared to ShF-DMEx1-SCM with 20 FFU/cell compared to 4.8 FFU/cell, respectively (Table 2). 

A similar experiment was carried out for virus production in SFM with cultures ShF-DMEx1-SFM and ShF-DMEx2-SFM. The different DME frequencies were applied from 2 dpcs. The ranges of glucose concentrations and pH values measured throughout the experiment were similar to those in SCM (Figure 4a,b, Table 2). 

In both cultures, peak total cell numbers of ~3 × 10^6^ cells/carrier were reached (Figure 4a,b). 

The cultures were maintained in SFM from 5 dpcs where ≥80% infection was seen. In ShF-DMEx1-SFM high-yield culture days were 7–11 dpcs with a peak titer of 6.8 log_10_ FFU/mL. ShF-DMEx2-SFM had one more high-yield culture day and a peak titer of 6.9 log_10_ FFU/mL (Figure 4c, Table 2). CSVY was ~1.5-fold higher in ShF-DMEx2-SFM as compared to ShF-DMEx1-SFM with 7.2 and 4.7 FFU/cell, respectively. Thus, two DME per day increased CSVY in cultures maintained in both SCM and SFM, although the difference was less pronounced under SF conditions.

### 3.6. Two DME Improved HCV Yields in SFM (CC-DMEx1-SFM_2_, CC-DMEx2-SFM)

Shake flask experiments suggested that applying two DME might enhance HCV yields in the CelCradle™. To evaluate this, cultures with one (CC-DMEx1-SFM_2_) and two DME (CC-DMEx2-SFM) were carried out in parallel. 

Cells were infected at a csGCR of 0.002 mmol/carrier/day at 2 dpcs. The different DME frequencies were applied from 4 dpcs. Glucose concentrations and pH values in both cultures were evaluated twice daily and adjusted when required.

**Figure 5:**
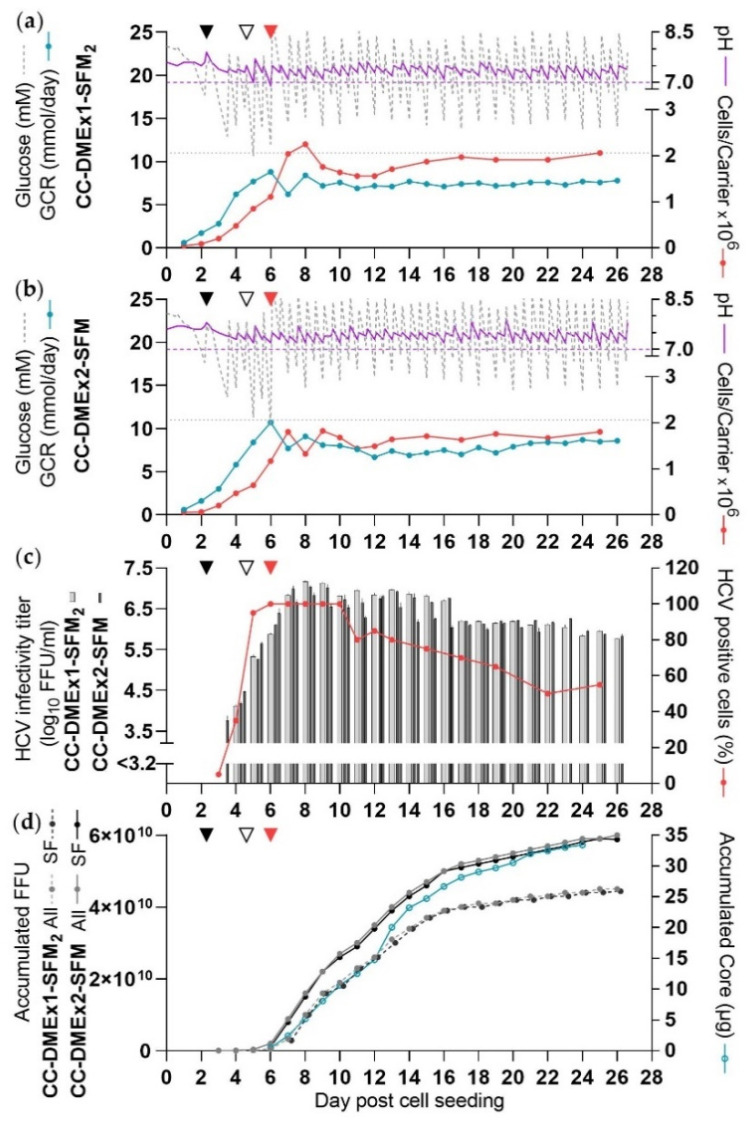
SF HCV production in the CelCradle™ with two DME (CC-DMEx1-SFM_2_ and CC-DMEx2-SFM). Two replicate CelCradle™ cultures were seeded with 2 × 10^5^ cells/carrier and infected as described for Figure 1 at 2 dpcs (black arrow). Purple dashed line: pH 7, grey dotted line: 11 mM glucose. At 3 dpcs medium was exchanged in the evening and from 4 dpcs medium was exchanged every morning in both cultures. Different DME frequencies, (**a**) CC-DMEx1-SFM_2_, morning versus (**b**) CC-DMEx2-SFM, morning and evening, were initiated at 4 dpcs. Glucose and/or NaHCO_3_ solution was added and glucose and pH were measured as described in Figure 1. Carriers were sampled for cell count every day until 13 dpcs and then less frequently. From 6 dpcs (morning) both cultures were maintained in SFM (red arrow). (**a**,**b**) Glucose concentration, pH, GCR, cells/carrier. (**c**) HCV infectivity titers and percentage HCV NS5A positive cells; the curve represents both cultures as identical values were recorded throughout the experiment. (**d**) Accumulated FFU calculated according to harvest volumes of 450 mL. Accumulated amounts of HCV Core protein from CC-DMEx2-SFM SF harvests until 24 dpcs.

In CC-DMEx1-SFM_2_ ranges of glucose concentrations and pH values were similar to those in previous experiments, CC-DMEx1-SCM and CC-DMEx1-SFM_1_ (Figure 5a, Table 1). Lactate levels peaked at 37 mM at 6 dpcs and subsequently decreased while the culture was maintained in SFM (Figure S3, Table 1). Concentrations of glutamine and ammonia were measured while the culture was maintained in SCM (containing GlutaMAX). Glutamine consumption peaked at 0.6 mmol/day at 6 dpcs and peak ammonia concentrations were around 1 mM prior to DME at 2, 3 and 6 dpcs (Figure S3).

In CC-DMEx2-SFM ranges of glucose concentrations and pH values were similar to that in CC-DMEx1-SFM_2_; however, pH levels were maintained >7 throughout the time of culture (Figure 5b, Table 1). Lactate levels peaked at lower levels than in CC-DMEx1-SFM_2_ (Figure S3, Table 1). When DME was carried out in both cultures in the mornings, lactate levels were generally 8–11 mM higher in CC-DMEx1-SFM_2_ compared to CC-DMEx2-SFM. In the evening when both cultures were sampled and DME was carried out in CC-DMEx2-SFM, lactate levels were similar (Figure S3). Concentrations of glutamine and ammonia were also similar to levels observed for CC-DMEx1-SFM_2_ (Figure S3, Table 1).

For both cultures, similar peak cell numbers of 1.8 × 10⁹ and 1.5 × 10⁹ were reached for one DME and two DME, respectively (Figure 5a,b, Table 1). 

Both cultures were estimated to be ≥80% infected from 5–15 dpcs and were maintained under SF conditions from 6 dpcs, already in the morning, with one or two harvests per day according to DME frequency. For both cultures there were 10 high-yield culture days on 7–16 dpcs (Figure 5c,d, Table 1). CC-DMEx1-SFM_2_ had moderate-yield culture days until 25 dpcs and a peak titer of 7.2 log_10_ FFU/mL at 8 dpcs. The total yield of virus in this culture until 25 dpcs, the last moderate-yield day, was 4.4 × 10^10^ FFU (Figure 5c, Table 1). CSVY was 24.5 FFU/cell (Table 1). CC-DMEx2-SFM had moderate-yield culture days until 26 dpcs, when the culture was terminated, and a peak titer of 7.0 log_10_ FFU/mL at 7 dpcs. The total yield of virus until the final day of culture, was 5.9 × 10^10^ FFU (Figure 5d, Table 1). CSVY was 41 FFU/cell (Table 1). In both cultures, virus harvests carried out after 16 dpcs did not contribute considerably to the total yield. 

The total yield of CC-DMEx2-SFM was ~34% higher than that of CC-DMEx1-SFM_2_ (Table 1). The total amount of HCV Core protein produced from CC-DMEx2-SFM by 24 dpcs was ~33 µg (Figure 5d).

### 3.7. Selected Characteristics Were Similar for HCV Produced in the CelCradle™ and in Monolayer Cell Cultures

As culture conditions in the CelCradle™ differ from those in standard monolayer cell culture, we compared selected characteristics of HCV produced in the CelCradle™ to HCV derived from T flasks.

**Figure 6:**
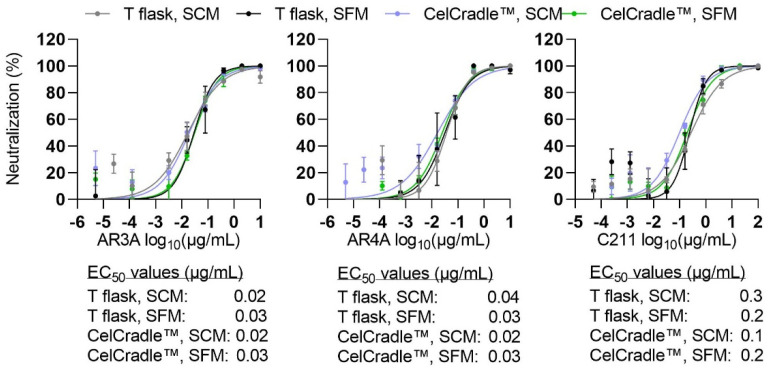
Neutralization sensitivity of HCV produced in the CelCradle™ and in monolayer cultures under SC and SF conditions is similar. Sensitivity to neutralization by human monoclonal antibodies AR3A (**left**) and AR4A (**middle**) and human polyclonal antibody preparation C211 (**right**) was compared for HCV produced under SC conditions (CC-DMEx1-SCM, Figure 1) or SF conditions (CC-DMEx1-SFM_1_, Figure 2) in the CelCradle™ or in T flasks in in vitro neutralization assays. Datapoints are means of triplicates with SEM. Variable slope sigmoidal dose-response curves were fitted to determine EC_50_ values as described in Materials and Methods; EC_50_ values are indicated in the figure.

Given the potential importance of the developed culture system for production of HCV for a whole virus vaccine, we first investigated the neutralization susceptibility of HCV produced in the CelCradle™ or T flasks under SC and SF conditions. Using the human polyclonal antibody preparation C211 [45] and two human monoclonal antibodies AR3A and AR4A targeting conformational epitopes in HCV E2 and E1E2, respectively [46,47], we found similar EC_50_ values for HCV produced under the four different conditions (Figure 6). Thus, HCV neutralization sensitivity was not altered for HCV produced in the CelCradle™. 

**Figure 7:**
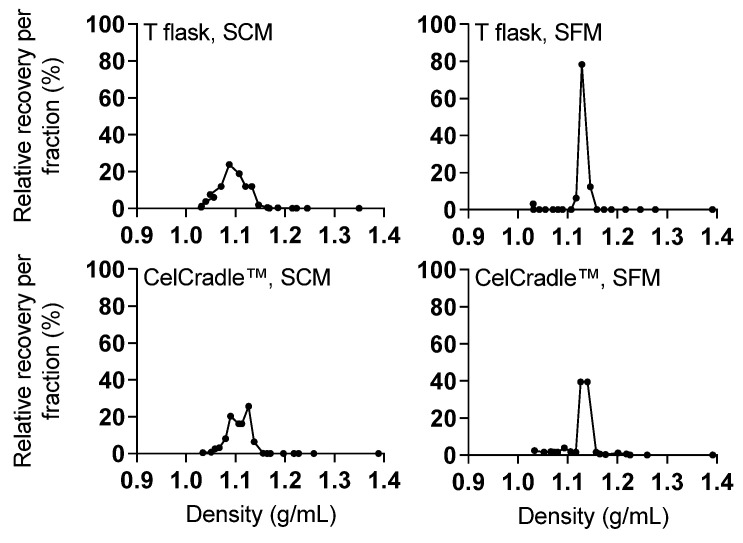
HCV produced in the CelCradle™ and in monolayer cultures showed similar buoyant density profiles. Buoyant density profiles were obtained for HCV produced in the CelCradle™ or T flasks under SC conditions (CC-DMEx1-SCM, Figure 1) and SF conditions (CC*-DMEx2-SFM, Figure 3) following fractionation by ultracentrifugation through a continuous 10–40% iodixanol gradient. The relative HCV recovery per fraction was calculated by relating amounts of HCV (FFU) in each fraction to the total amount of HCV in all fractions.

In addition, culture conditions have been described to have an effect on buoyant density of the virus particles. HCV particles from patients display varying densities, with greater infectivity of low-density viruses [51]. In a previous study, HCV from SF monolayer cultures displayed a narrow density peak, while HCV derived from SC monolayer cultures had a broad density distribution [39]. In line with these findings, under SC conditions a broad density distribution was observed with 78% of T flask- and 86% of CelCradle™-derived infectious HCV in the density range 1.07–1.13 g/mL (Figure 6). Under SF conditions a homogeneous density peak was observed with 91% of T flask- and 79% of CelCradle™-derived infectious HCV in the density range 1.13–1.15 g/mL (Figure 7). Thus, while we confirmed the influence of serum on the buoyant density profile, HCV produced in the CelCradle™ had similar buoyant density profiles as HCV produced in T flasks.

We found that HCV produced in the CelCradle™ had slightly increased specific infectivity calculated based on infectivity and RNA titers compared to HCV produced in monolayer cultures. Analyzing selected harvests, the specific infectivity of HCV produced in the CelCradle™ under SC and SF conditions was in the range of 0.04–0.2 FFU/IU and 0.07–0.8 FFU/IU, respectively (Figure S4). For comparison, the specific infectivity reported for T flask-derived virus from SC or SF cultures was 0.03 FFU/IU and 0.08 FFU/IU, respectively [32]. As reported for monolayer cultures [32], we observed a slightly higher specific infectivity for HCV produced under SF versus SC conditions in the CelCradle™.

Overall, HCV produced in SCM and SFM in the CelCradle™ showed similar characteristics as HCV produced in SCM and SFM in T flasks.

Furthermore, we investigated the influence of the relatively long culture time in the CelCradle™ on HCV genetic stability. Sanger sequencing did not indicate any evidence for acquisition of mutations in the open reading frame of HCV from a harvest collected at 21 dpcs of a SF production (CC*-DMEx2-SFM, Figure 3).

## 4. Discussion

In this study, efficient SF HCV production in adherent Huh7.5 cells was established in the laboratory-scale CelCradle™ packed-bed bioreactor, a tide-motion culture system also available for large-scale GMP production. The time of infection was adjusted to achieve simultaneous cell expansion and infection spread, which was expected to be important for virus yields. Doubling the number of carriers in the culture reduced CSVY and total virus yield, whereas increasing the harvest frequency to two harvests instead of one per day resulted in improved virus yields.

Cell numbers in the CelCradle™ cultures exceeded 1 × 10^6^ cells/carrier at 6 dpcs in all cultures and peak total cell numbers were in the range of 0.96–1.8 × 10^9^ cells in cultures with 5.5 g BioNOCII™ carriers. This is comparable to what has previously been seen for Huh7 cells in a CelCradle™ culture [34]. There was a trend for glucose consumption to reflect changes in cell numbers, but there was no linear correlation. GCR values peaked while the cultures were maintained in SCM and declined following the change to SF conditions. This might be due to altered cell metabolism in the different medium composition. According to previous experience glutamine was not added in SF conditions [39]. An exception was the culture with increased carrier concentration (CC*-DMEx2-SFM), where GlutaMAX was supplied under SF conditions demonstrating low consumption. 

Cultures were initiated in cell culture medium containing serum, required for Huh7.5 cell attachment, as well as for efficient HCV infection and infection spread [unpublished data, [39]]. In monolayer cultures, applying SF conditions already at a low level of infection delayed or inhibited the spread of infection, while maintaining fully infected cultures under SF conditions seemed to delay cell death [39]. Ideally, biologicals for human use should be produced without animal components; however, GMP-grade serum complying with specific regulatory guidelines may be used, which would be highly relevant for this application [52].

Compared to viruses such as influenza A virus and SARS-CoV-2, HCV spreads relatively slowly in cell culture and is characterized by lower infectivity titers [24,36,53,54]. Thus, to align peak in cell numbers with peak in the percentage of HCV infection, and to limit the amount of virus needed for inoculation, cultures were inoculated at csGCR of 0.002 mmol/carrier/day. This resulted in ≥80% of culture cells being infected when cell numbers were around 1 × 10^6^ cells/per carrier, typically 4 days post inoculation. The first high-yield culture day in the CelCradle™ cultures was two days after the observation of 80% infection. It was thus critical to apply SF conditions before this timepoint. A combination of fed-batch culture mode and medium exchange was applied in this study rather than testing batch production due to the slow spread of infection. Further, relatively high HCV infectivity titers were obtained for an extended period of time; however, the percentage of HCV infected cells and HCV infectivity titers declined with time, similarly to what has been observed for HCV infected monolayer cultures [39,41,55,56]. Although the cell death induced by most HCV recombinants in Huh7.5 cells is much less pronounced than the cytopathic effect seen in vitro for viruses such as influenza A virus and SARS-CoV-2, it may in part explain the observed decline in infectivity titer [36,41,53,57]. In addition, it has been observed in HCV infected monolayer cultures that upon infection induced cell death, a population of infection resistant cells may expand to dominate the culture [55,56]. This phenomenon might further contribute to the observed decline in infectivity titers in the cultures.

The highest HCV virus yield obtained from the CelCradle™ in this study was obtained from a culture with standard carrier concentrations maintained with two DME (virus harvests) per day (CC-DMEx2-SFM). The CSVY of this culture was ~41 FFU/cell, which is comparable to the productivity observed for influenza A virus in Madin–Darby canine kidney cells cultured in the CelCradle™ run in perfusion mode in another study [35]. The total yield of accumulated FFU was increased by ~34% when compared to a culture maintained in parallel with one DME and harvest. This was similar to observations in shake flask cultures where two as compared to one DME improved CSVY by ~4-fold and ~1.5-fold under SC and SF conditions, respectively. The more pronounced effect observed for shake flask cultures under SC conditions could be due to the higher concentrations of waste products reached in these cultures. Indeed, for SC shake flasks there was a trend for slightly higher concentrations of ammonia with only one as compared to two DME. Comparing one and two DME and harvests, the shake flask and CelCradle™ cultures maintained with only one DME reached higher concentrations of lactate, and pH values were more difficult to control. While details about the effect of lactate on Huh7.5 cells have not been reported, levels of up to 50 mM were measured in Huh7 cells in a previous study [58]. Although lactate concentrations observed in this study did not seem to influence cell numbers, this factor could potentially contribute to reduced virus yields [59]. Concentrations of other inhibitory factors or limiting nutrients not evaluated in this study might similarly influence yields. While the shake flask cultures proved useful for evaluation of medium exchange frequency in this study, evaluation of neutralization sensitivity, buoyant density, and specific infectivity of virus harvested from these cultures would be relevant to further characterize this scale-down model.

Increasing carrier concentration and total cell numbers (CC*-DMEx2-SFM) resulted in a decreased virus production yield. Such cell density effects might be due to shortage of nutrients or accumulation of waste products. Thus, in CC*-DMEx2-SFM higher lactate concentrations were measured than in other experiments [60,61]. Certainly, the difference in virus yields between CC-DMEx1-SFM_2_ and CC-DMEx2-SFM discussed above might indicate that reducing the concentration of waste products, e.g., by additional medium exchanges, perfusion- or recirculation culture mode, could mitigate the negative effect of the high cell numbers. This is in line with findings in other studies indicating the relevance of optimizing feeding strategies to improve cell culture yields [35,62]. The relatively low stability of HCV at 37 °C in SFM might indeed favor a feeding strategy based on frequent or continuous harvesting [39]. Virus yield from cultures with high cell densities might also be improved by optimizing cell culture medium composition [60,63]. Finally, generation and application of a cell bank derived from a high producer cell clone would be an attractive approach to further standardize and optimize virus production.

HCV sensitivity to neutralizing antibodies indicates that relevant target epitopes are accessible on the virus particle. Importantly, sensitivity to neutralization by human monoclonal antibodies AR3A and AR4A [46,47,64], which target epitopes associated with protection from chronic infection in humans [20], as well as by the polyclonal antibody preparation C211 [45], were similar, comparing T flask- and CelCradle™-derived HCV. This indicated that the mode of HCV production did not confer changes to those highly relevant epitopes. HCV particles are associated with lipoproteins and the composition of the lipo-viro-particle affects the buoyant density of the HCV particles [65]. The association with lipoproteins has been shown to influence neutralization susceptibility as well as infectivity [51,66,67] and could thus potentially influence virus immunogenicity. In accordance with what was previously described [39], the buoyant density profiles were different for HCV derived from SCM and SFM; however, buoyant density profiles were similar for T flask- and CelCradle™-derived HCV. These observations are in line with findings in a previous study describing HCV production in a small-scale hollow fiber bioreactor [32]. The hollow fiber bioreactor had a working volume of 20 mL and production yields were up to 2.4 × 10^9^ FFU from a total harvest volume of 260 mL in a 40-day culture [32], ~25-fold less than in CC-DMEx2-SFM. While HCV infectivity titers of up to 7.6 log_10_ FFU/mL were obtained in the hollow fiber bioreactor, this bioreactor cannot be scaled for production purposes [32]. 

Only a few other studies report HCV production in small scale bioreactors, where HCV infectivity titers of up to 5 log_10_ FFU/mL were achieved in Huh7 cells grown on microcarriers [68,69]. HCV can also be produced in large scale monolayer systems such as the Cell Factory system (Nunc) or the CellSTACK system (Corning) [19,70]; however, drawbacks of these systems include limited scalability and suboptimal control of cell culture parameters, such as pH and gas supply [31]. Furthermore, large-scale monolayer systems generally require extensive manual handling, whereas bioreactors such as the TideXCell systems, the large scale versions of the CelCradle™, are highly automated systems with closed-loop pH control and programmable feeding or perfusion of cell culture medium [31,38]. The yield of infectious virus obtained from CC-DMEx2-SFM was 5–10 fold higher than yields typically obtained from HCV production in Cell Factory systems [70]. The largest available TideXCell system (Esco Aster Pte. Ltd.) can hold almost 1000 times more BioNOCII™ carriers than the CelCradle™. Interestingly, efficient scale-up of influenza A virus production from the CelCradle™ to the 10 L TideCell002 (with 10 times more BioNOCII™ carriers than the CelCradle™) was recently demonstrated [35]. Assuming similar scalability for HCV production, TideXCell bioreactors are promising systems for large-scale HCV production.

While proof-of-concept studies have demonstrated induction of neutralizing antibodies upon immunization of mice and non-human primates with inactivated whole HCV particles [19,71], the appropriate vaccine dose remains to be defined. However, assuming a dose of 10^7^ FFU and a recovery of 50% during the downstream purification process [70], the yield from the CC-DMEx2-SFM would be equivalent to around 2900 doses. Assuming a linear scalability to the TideXCell system, one culture might yield several hundred thousand doses. Although more studies are needed to address dose requirements for optimal induction of neutralizing antibody responses, this study highlights the applicability of the CelCradle™ laboratory-scale packed-bed bioreactor for animal immunogenicity studies, and the potential suitability of the TideXCell systems for larger-scale production. 

## Figures and Tables

**Table 1:** Overview of cultivation parameters, rates, and yields of CelCradle™ experiments.

Experiment	Cells Only SCM	CC-DMEx1-SCM	CC-DMEx1-SFM_1_	CC*-DMEx2-SFM	CC-DMEx1-SFM_2_	CC-DMEx2-SFM
SF virus production ^a^	na	no	yes	yes	yes	yes
DME/harvests per day	1	1	1	2	1 ^b^	2 ^b^
BioNOCII™ carriers, g	5.5	5.5	5.5	10.7	5.5	5.5
Peak cell numbers						
Day of peak total cell number, dpcs	9	8	8	12	8	7
Peak total cell number	1.6 × 10^9^	0.96 × 10^9^	1.2 × 10^9^	3.6 × 10^9^	1.8 × 10^9^	1.5 × 10^9^
cells/mL	3.2 × 10^6^	1.9 × 10^6^	2.4 × 10^6^	7.1 × 10^6^	3.6 × 10^6^	2.9 × 10^6^
cells/carrier	2.0 × 10^6^	1.2 × 10^6^	1.5 × 10^6^	2.3 × 10^6^	2.3 × 10^6^	1.8 × 10^6^
Cell culture supernatant parameters						
Peak GCR, mmol/day (dpcs)	9 (8)	10 (6)	11 (7)	23 (7)	9 (6)	11 (6)
Glucose range, mM	5–24	10–25	11–25	5–29	11–27	10–27
Lactate peak concentration, mM (dpcs) ^c^	nd	nd	nd	48 (7)	37 (6)	26 (6)
Glutamine range, mM ^c^	nd	nd	nd	1.7–4.3	2.1–3.6	2.7–3.6
Ammonia range, mM ^c^	nd	nd	nd	0.3–1.0	0.3–1.0	0.3–1.0
pH range	6.8–8.3	6.9–7.7	6.9–8.3	6.9–7.9	6.9–7.9	7.1–7.9
Virus yield						
Total harvest volume, L ^d^	na	2.3	5.9	10.6	8.6	18.5
Peak infectivity titer, log_10_ FFU/mL	na	6.8	6.9	6.9	7.2	7.0
Days with high- or moderate yield	na	9–13	7–19	8–18 ^e^	7–25	6–26 ^f^
Total accumulated FFU ^g^	na	0.81 × 10^10^	2.2 × 10^10^	1.7 × 10^10^	4.4 × 10^10^	5.9 × 10^10^
CSVY, FFU/cell	na	8.5	18.5	4.8	24.5	41.0
Yield compared to CC-DMEx2-SFM ^h^	na	0.14	0.37	0.29	0.74	1.0

CC: CelCradle™, DME: daily medium exchange, SCM: serum-containing medium, SFM: serum-free medium, na: not applicable, dpcs: day post cell seeding, GCR: glucose consumption rate, nd: not determined, FFU: focus forming unit(s), CSVY: cell specific virus yield. ^a^ Both SC and SF virus production cultures were initiated in SCM (cell seed and infection) as described in Materials and Methods. ^b^ CC-DMEx1-SFM_2_ and CC-DMEx2-SFM were done in parallel; concerning culture conditions, CC-DMEx1-SFM_2_ is a replicate of CC-DMEx1-SFM_1_. ^c^ Lactate-, glutamine- and ammonia concentrations evaluated in selected experiments. In most experiments, glutamine (supplied as GlutaMAX) was only supplied in SCM. ^d^ The total harvest volume of high- and moderate yield culture days. The volume of each harvest was ~0.45 L. ^e^ The harvest at 7 dpcs was not in SFM and is not included. ^f^ Last day of culture. ^g^ The sum of the total number of FFU obtained from high- and moderate yield culture days in SFM in each experiment. In experiments without a SF production phase, the number represents harvests in SC conditions. ^h^ Calculated from the total accumulated FFU.

**Table 2:** Overview of cultivation parameters, rates, and yields of shake flask experiments.

Culture	ShF-DMEx1-SCM	ShF-DMEx2-SCM	ShF-DMEx1-SFM	ShF-DMEx2-SFM
SF virus production ^a^	no	no	yes	yes
DME/harvests per day	1	2	1	2
BioNOCII™ carriers, g	0.39	0.39	0.39	0.39
Peak cell numbers				
Day of peak total cell number, dpcs	5	6	9	7
Peak total cell number	1.1 × 10^8^	0.94 × 10^8^	0.95× 10^8^	1.4 × 10^8^
cells/mL	3.9 × 10^6^	3.5 × 10^6^	4.9 × 10^6^	5.5 × 10^6^
cells/carrier	2.3 × 10^6^	2.1 × 10^6^	2.9 × 10^6^	3.2 × 10^6^
Cell culture supernatant				
Peak csGCR, mmol/carrier/day (dpcs)	0.014 (6)	0.017 (6)	0.016 (6)	0.019 (5)
Glucose range, mM	12–31	7–32	11–32	9–34
Lactate peak concentration, mM (dpcs)	43 (8)	33 (7)	39 (5)	36 (5)
Glutamine range, mM ^b^	1.2–3.6	1.8–4.0	1.7–3.6	2.0–3.6
Ammonia range, mM ^b^	0.4–1.5	0.3–1.4	0.3–1.2	0.3–1.0
pH range	6.8–8.1	6.9–8.1	6.7–7.8	6.8–8.1
Virus yield				
Peak infectivity titer, log_10_ FFU/mL	6.9	7.1	6.8	6.9
Days with high- or moderate yield	7–12 ^c^	5–12 ^c^	7–11 ^c^	6–11 ^c^
CSVY, FFU/cell	4.8	20.0	4.7	7.2

ShF: shake flask, DME: daily medium exchange, SCM: serum-containing medium, SFM: serum-free medium, dpcs: day post cell seeding, csGCR: carrier specific glucose consumption rate, FFU: focus forming unit(s), CSVY: cell specific virus yield. ^a^ Both SC and SF virus production cultures were initiated in SCM (cell seeding and infection), as described in Materials and Methods. ^b^ Glutamine was only supplied in SCM. ^c^ Last day of culture.

## Data Availability

Not applicable.

## Supplementary Materials

The following supporting information can be downloaded at: https://www.mdpi.com/article/10.3390/vaccines10020249/s1, Figure S1: Cell attachment to BioNOCII™ carriers in CelCradle™ cultures, Figure S2: Cell culture supernatant parameters of CC*-DMEx2-SFM with 10.7 g BioNOCII™ carriers, Figure S3: Cell culture supernatant parameters of CC-DMEx1-SFM_2_ and CC-DMEx2-SFM comparing one and two daily medium exchanges, Figure S4: HCV produced in the CelCradle™ showed a slight increase in specific infectivity compared to HCV produced in monolayer cultures.
