# Supplementary material for: High-Titer Hepatitis C Virus Production in a Scalable Single-Use High Cell Density Bioreactor
Source: Vaccines (Basel). 2022 Feb 7;10(2):249. doi: 10.3390/vaccines10020249 (PMC8880717; doi:10.3390/vaccines10020249)
Supplement: Supplementary file 1 [file vaccines-10-00249-s001.zip › vaccines-1541518-supplementary.pdf]

Supplementary Materials

High-titer hepatitis C virus production in a scalable single-use high cell density bioreactor

Anna Offersgaard[1,2], Carlos Rene Duarte Hernandez[1,2], Anne Finne Pihl[1,2], Nandini Prabhakar Venkatesan[3], Henrik Krarup[4], Xiangliang Lin[3], Udo Reichl[5], Jens Bukh[1,2], Yvonne Genzel[5], Judith Margarete Gottwein[1,2*]

[1] Copenhagen Hepatitis C Program (CO-HEP), Department of Infectious Diseases, Copenhagen University Hospital–Hvidovre, 2650 Hvidovre, Denmark; anna.offersgaard@regionh.dk (A.O.); carlos.rene.duarte.hernandez@regionh.dk (C.R.D.H.); anne.finne.pihl@regionh.dk (A.F.P.); jbukh@sund.ku.dk (J.B.); jgottwein@sund.ku.dk (J.M.G.)

[2] Copenhagen Hepatitis C Program (CO-HEP), Department of Immunology and Microbiology, Faculty of Health and Medical Sciences, University of Copenhagen, 2200 Copenhagen, Denmark

[3] Esco Aster Pte Ltd., Singapore 486 777, Singapore; nandini.prabhakar@escoaster.com (N.P.V.); xl.lin@escoaster.com (X.L.)

[4] Department of Molecular Diagnostics, Aalborg University Hospital, Aalborg, Denmark; h.krarup@rn.dk

[5] Bioprocess Engineering, Max Planck Institute for Dynamics of Complex Technical Systems, 39106 Magdeburg, Germany; ureichl@mpi-magdeburg.mpg.de (U.R.); genzel@mpi-magdeburg.mpg.de (Y.G.)

* Correspondence: jgottwein@sund.ku.dk (J.M.G.)

Supplementary Figures

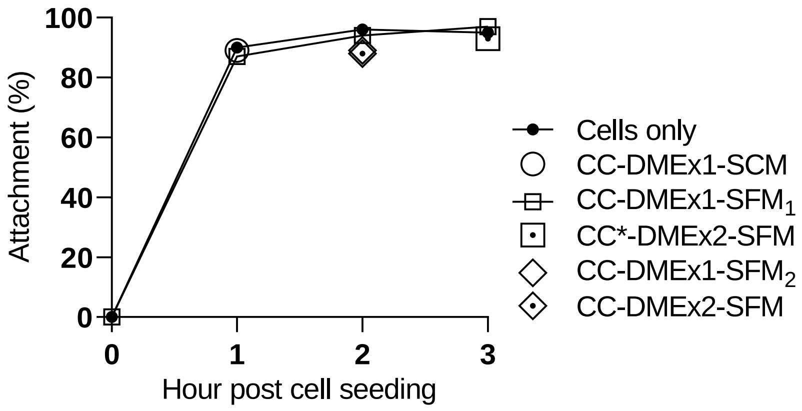

Figure S1. Cell attachment to BioNOCII™ carriers in CelCradle™ cultures. Cultures were seeded with around 2 x 10^5 cells/carrier. Cells in suspension were sampled to calculate percentage attachment efficiency as described in Materials and Methods.

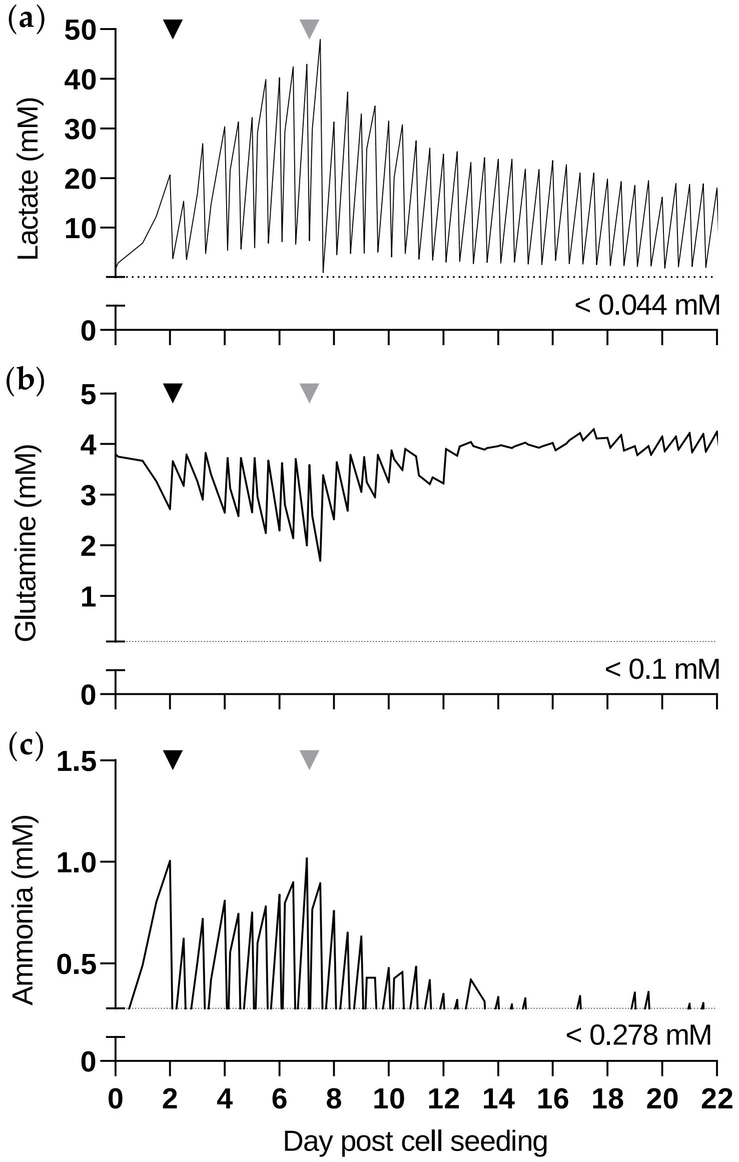

Figure S2. Cell culture supernatant parameters of CC*-DMEx2-SFM with 10.7 g BioNOCII™ carriers. (**a**) Lactate, (**b**) glutamine and (**c**) ammonia concentrations were measured upon medium sampling in the experiment shown in Figure 3 (Table 1, CC*-DMEx2-SFM). The culture medium during the serum-free (SF) production phase in this experiment contained 4mM GlutaMAX. The lower limit of detection of each assay is indicated. Time of infection (black arrow) and time of change to serum-free medium (SFM) (grey arrow) are indicated.

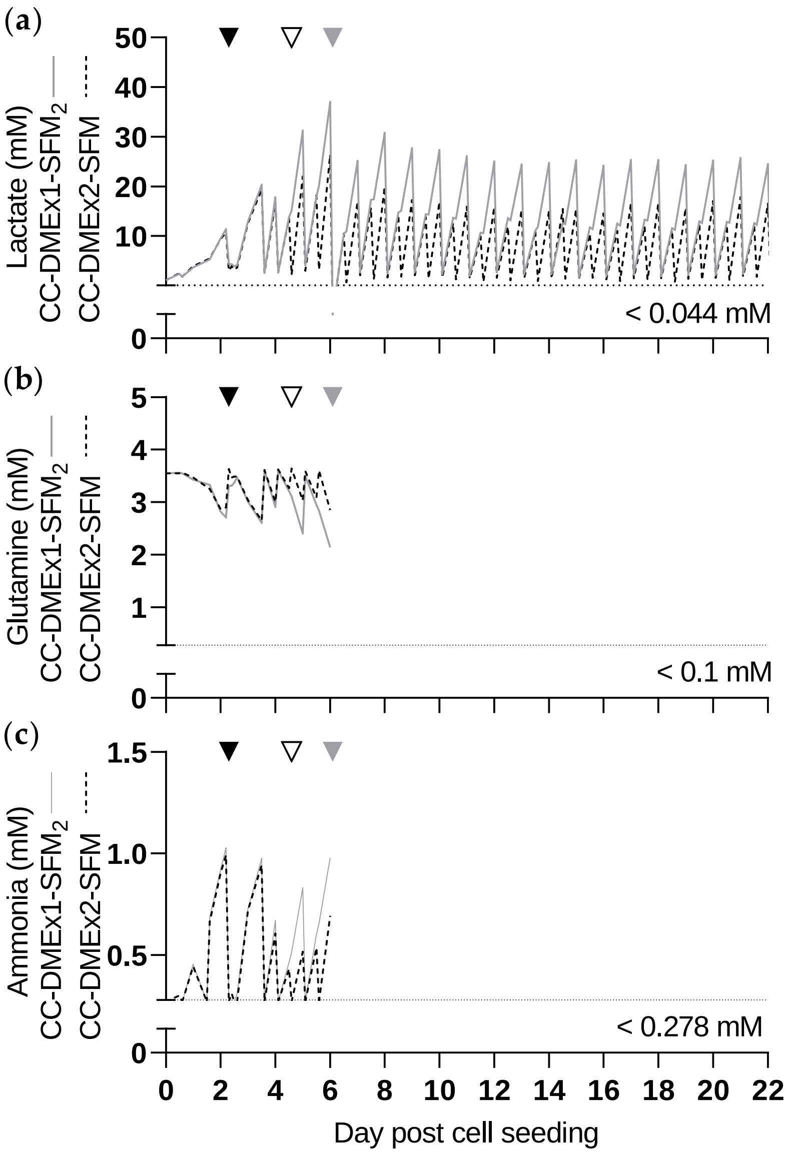

Figure S3. Cell culture supernatant parameters of CC-DMEx1-SFM_2 and CC-DMEx2-SFM comparing one and two daily medium exchanges. (**a**) Lactate, (**b**) glutamine and (**c**) ammonia concentrations were measured upon medium sampling in the experiment shown in Figure 5 (Table 1, CC-DMEx1-SFM_2 and CC-DMEx2-SFM). No glutamine was added in the SFM, thus, levels of glutamine and ammonia were only measured during cultivation in serum-containing medium (SCM). The lower limit of detection of each assay is indicated. Time of infection (black arrow), time of initiation of different medium exchange frequencies (open arrow), and time of change to SFM (grey arrow) are indicated.

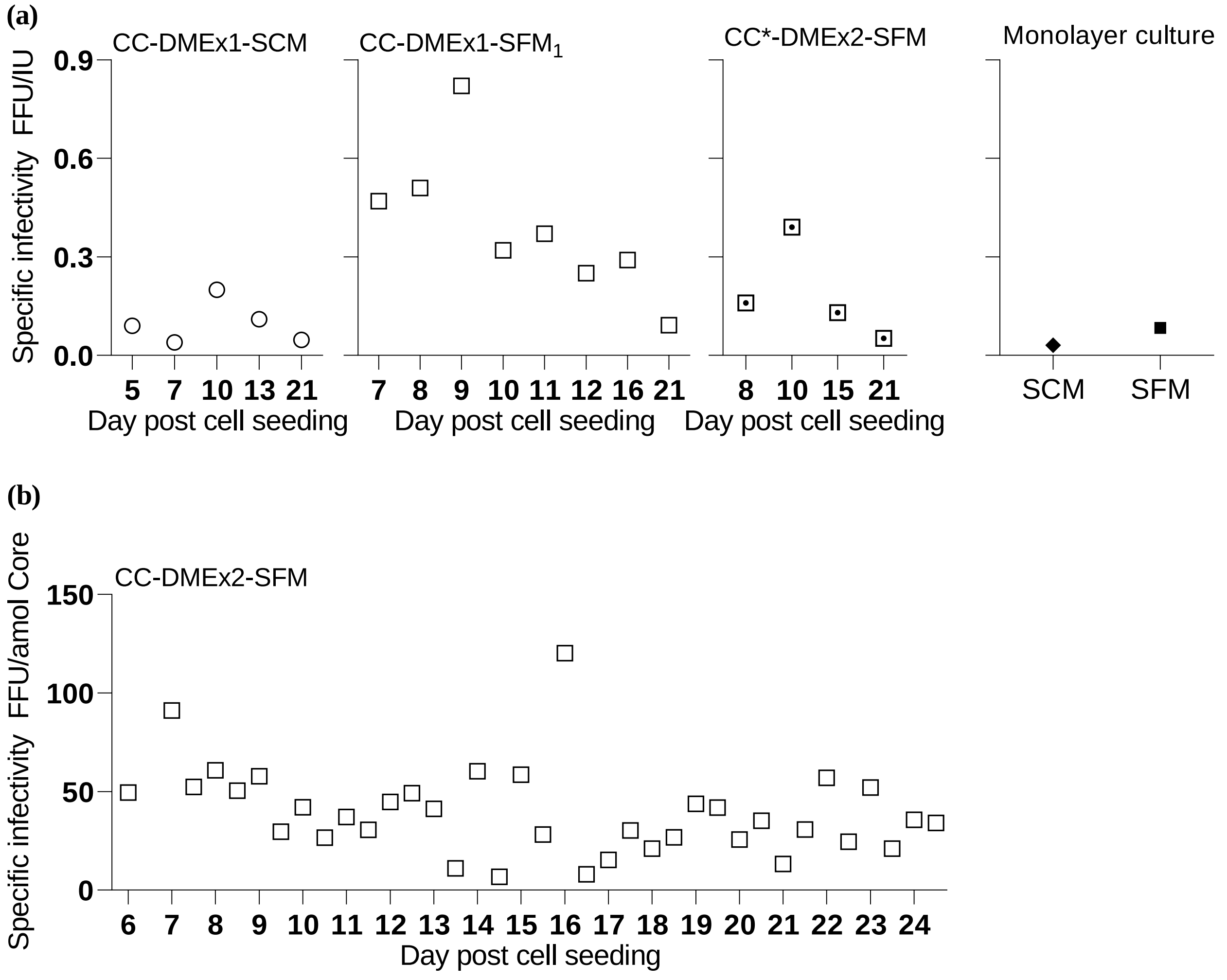

Figure S4. HCV produced in the CelCradle™ showed a slight increase in specific infectivity compared to HCV produced in monolayer cultures. (**a**) The specific infectivity (FFU/IU) was determined for several harvests derived from different CelCradle™ cultures (individual dots). Data points from CelCradle™ cultures represent CC-DMEx1-SCM (harvests from 5, 7, 10, 13, and 21 dpcs) (Figure 1) cultivated with SCM, CC-DMEx1-SFM$_1$ (harvests from 7, 8, 9, 10, 11, 12, 16, and 21 dpcs) (Figure 2), and CC*-DMEx2-SFM (harvests from 8, 10, 15, and 21 dpcs) (Figure 3), both cultivated with SFM. The specific infectivity of HCV derived from monolayer cultures and harvested in SCM or SFM were previously reported and are reproduced for comparison [1]. (**b**) The specific infectivity (FFU/amol Core) was determined for serum-free harvests from the experiment CC-DMEx2-SFM (Figure 5b-d).

References

1. Pihl, A.F.; Offersgaard, A.F.; Mathiesen, C.K.; Prentoe, J.; Fahnoe, U.; Krarup, H.; Bukh, J.; Gottwein, J.M. High density Huh7.5 cell hollow fiber bioreactor culture for high-yield production of hepatitis C virus and studies of antivirals. *Sci Rep* **2018**, *8*, 17505, doi:10.1038/s41598-018-35010-5.
